# A Pilot Study on Monomer and Bisphenol A (BPA) Release from UDMA-Based and Conventional Indirect Veneering Composites

**DOI:** 10.3390/polym14214580

**Published:** 2022-10-28

**Authors:** Tristan Hampe, Julia Liersch, Bernhard Wiechens, Torsten Wassmann, Andrea Schubert, Mohammad Alhussein, Ralf Bürgers, Sebastian Krohn

**Affiliations:** 1 Department of Prosthodontics, University Medical Center Göttingen, 37075 Göttingen, Germany; 2 Department of Orthodontics, University Medical Center Göttingen, 37075 Göttingen, Germany; 3 Molecular Phytopathology and Mycotoxin Research, University of Göttingen, 37077 Göttingen, Germany; 4 Department of Orthodontics, University Hospital Regensburg, 93053 Regensburg, Germany

**Keywords:** materials testing, dental materials, indirect composite, urethane dimethacrylate, bisphenol A, bisphenol A-glycidyl methacrylate

## Abstract

This study aimed to investigate the release of common monomers from conventional (Dialog Vario, Enamel Plus HFO) and UDMA-based indirect veneering composites (VITA VM LC, GC Gradia). Ten cylindrical samples of each material were prepared (*n* = 40), immersed in HPLC grade water, and incubated for 24 h in an incubation shaker at 37 °C and 112 rpm. Extraction was performed following ISO 10993-12 and monomers were detected and quantified by HPLC-MS/MS. In all the samples, urethane dimethacrylate (UDMA) and bisphenol A (BPA) were quantifiable. Compared to water blanks, BPA levels were only elevated in the eluates from conventional composites. In all other samples, concentrations were in the range of extraneous BPA and were therefore clinically irrelevant. Low concentrations of Bisphenol A-glycidyl methacrylate (BisGMA) were found in one BPA-free composite and in both conventional materials. Statistical analyses showed that BPA-free materials released significantly less BisGMA and no BPA, while UDMA elution was comparable to elution from conventional materials. All measured concentrations were below reported effective cytotoxic concentrations. Considering these results, the substitution of BPA-derivatives with UDMA might be beneficial since BPA-associated adverse effects are ruled out. Further studies should be enrolled to test the biocompatibility of UDMA on cells of the oral environment.

## 1. Introduction

Improvements in mechanical properties and higher esthetic demands led to the application of composites in various areas of dentistry [1,2]. Of these, indirect composites are used in dental laboratories for the fabrication of tooth-colored restorations or as a veneering material for removable and fixed dental prostheses [3,4,5]. In general, the composition of indirect veneering composites is very similar to that of direct restorative resins [6]. Composites are a complex class of organic/inorganic hybrid materials, in which an organic polymer matrix is bonded with inorganic fillers by silane coupling agents [7,8]. During polymerization, monomers of the organic matrix are crosslinked and form a rigid polymer network [9]. However, propagating crosslinking restricts diffusion, and thus, full conversion of all present monomers is hindered [9]. Incomplete conversion results in residual unreacted monomers, which are known to leach into surrounding aqueous phases [10]. It is suggested that excess monomers might present cytotoxic [11,12,13,14,15,16,17], genotoxic [18,19], allergenic [20,21], and/or estrogenic potential [22,23]. In recent years, the release of residual monomers and other substances, like bisphenol A (BPA) from dental composites, has been a cause of public concern [10]. However, only BPA derivatives are utilized in dentistry and not pure BPA and thus, only small amounts are leachable, which might be due to BPA contaminations during manufacturing [24,25]. As a result, some manufacturers have substituted BPA derivatives, like bisphenol A-glycidyl methacrylate (BisGMA), with urethane dimethacrylate (UDMA) and introduced UDMA-based composites to avoid the release of BPA and its derivatives [26,27,28]. In a preceding work, we found that BPA-free temporary crown and bridge materials release significantly more UDMA, but not BisGMA, in comparison to conventional materials [29]. As these materials were introduced as being more biocompatible, the substitution of BisGMA needs further investigation.

There are numerous studies on the release of monomers from direct restorative composites [30,31,32,33,34,35,36], but surprisingly there is a lack of investigations on the monomer elution from indirect veneering composites. In contrast to direct restorative composites, indirect veneering composites are used in higher amounts and therefore larger surface areas interact with the oral environment. Since monomer release depends on the extraction ratio (surface area/solvent) [37,38,39], larger surface areas could aggravate biocompatibility concerns. However, indirect composites usually show more complete polymerization due to better curing methods outside the oral cavity [40] and might therefore elute lower amounts of substances from the polymer matrix. Thus, the present pilot study aimed to investigate the release of common monomers and BPA from indirect veneering composites in order to compare the elution pattern between conventional and UDMA-based materials.

## 2. Materials and Methods

### 2.1. Sample Preparation

According to the manufacturer’s specifications standardized cylindrical samples of two conventional veneering composites, Dialog Vario (Schütz Dental, Rosbach, Germany) and Enamel Plus HFO (Micerium, Avegno, Italy), as well as two UDMA-based veneering composites VITA VM LC (VITA Zahnfabrik, Bad Säckingen, Germany) and GC Gradia (GC Corporation, Tokyo, Japan) were prepared. Information about the composition of the veneering composites is given in Table 1.

The uncured composite was placed in polytetrafluorethylene (PTFE) molds (inner diameter: 10 mm; height 7 mm) using sterile composite instruments. Composite insertion was made in 2 mm increments, as specified by the manufacturers. The samples were cured in a high-performance laboratory light-curing unit with a wavelength range of 390–540 nm (HiLite Power, Heraeus Kulzer, Hanau, Germany) at 200 W for 180 s. To prevent the formation of an oxygen inhibition layer, the last increment was covered with a glycerine-based masking gel (SR Gel, Ivoclar Vivadent, Liechtenstein). After preparation, the gel was removed by cleaning the samples with HPLC-grade water (Honeywell International, Charlotte, NC, USA). This resulted in 10 samples of each material (*n* = 40) with a surface area of 3.77 cm^2^.

### 2.2. Incubation

UV-protected borosilicate vials with PTFE-coated closures were used for sample incubation. Each vial was cleaned using HPLC-grade methanol (Sigma Aldrich, Munich, Germany) and residual methanol was removed by rinsing with HPLC-grade water twice. All vials were sterilized by autoclaving at 121 °C for 20 min (Systec VL-95, Systec, Lindau, Germany). Subsequently, the vials were dried in a laboratory oven (Thermo Scientific Oven T20, Thermo Fisher Scientific, Waltham, MA, USA) at 60 °C for 18 h.

Two samples per vial were immersed in 2.8 mL of HPLC grade water. The extraction ratio (surface area/solvent volume) was chosen following ISO 10993-12 and all samples were fully covered with HPLC grade water. Incubation was performed in an incubator shaker (Excella E24; New Brunswick Scientific, Enfield, CT, USA) for 24 h at 37 °C and 112 rpm. The rotation frequency was chosen to simulate intraoral agitation and to ensure the immersion of all sample surfaces. Following incubation, the samples were removed, and to prevent secondary chemical reactions, the eluate was frozen at −18 °C and kept in the dark.

### 2.3. HPLC-MS/MS Analysis

Immediately before analyses, all samples were thawed at room temperature and dried using a speed vacuum concentrator RVC 2-25 CD plus (Christ, Osterode am Harz, Germany) at 37 °C. Dry samples were dissolved in 2 mL methanol. For HPLC-MS/MS analysis, each sample was divided, and an aliquot of 1 mL was transferred to a separate HPLC amber glass vial. One half was utilized for the derivatization and analysis of BPA and the other half was used for the detection and quantification of BisGMA and UDMA.

**Figure 1:**
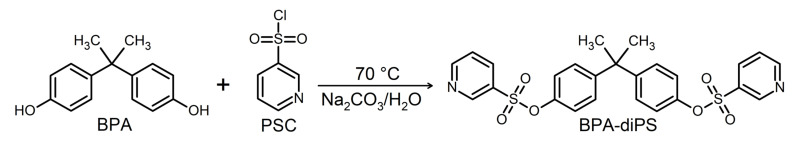
Derivatization of bisphenol A (BPA) with pyridine-3-sulfonylchloride (PSC). BPA diPS, bisphenol A derivatized with pyridine-3-sulfonyl chloride.

BPA elutes were analyzed immediately after derivatization of BPA using pyridine-3-sulfonyl chloride, as described by Regueiro et al. [41]. The derivatization scheme is shown in Figure 1.

Isotopically labeled d16BPA was used as an internal standard. The chemicals used for derivatization and HPLC analysis are listed in Table 2.

HPLC-MS/MS analysis was performed using an Agilent 1290 Infinity II HPLC system (Agilent Technologies, Palo Alto, CA, USA) coupled with an Agilent 6460 triple quadrupole detector (Agilent Technologies, Palo Alto, CA, USA). Separation was performed using a Polaris 3 C18-Ether column, 2 × 100 mm with a 3 µm particle size (Agilent Technologies, Palo Alto, CA, USA).

The column was kept at 40 °C, and the injection volume was 7 µL for BPA and 6 µL for UDMA and BisGMA analyses. For BPA, UDMA and BisGMA analysis solvent A was water with 0.1% formic acid [*v*/*v*], and solvent B was methanol with 0.1% formic acid in [*v*/*v*]. The gradient was as follows: 0 to 0.2 min, 30% B; 0.2 to 6 min, 30% to 98% B; 6 to 10 min, 98% B; 10 to 10.50 min, 98% to 30% B; and 10.50 to 14 min, 30%. The eluent was ionized using an ESI source. Nebulizer pressure was 60 psi, and the capillary voltage was 4000 V. The inert gas was nitrogen at 350 °C with a flow rate of 13 l/min. Derivatized BPA (dBPA) was quantified using multiple reactions monitoring (MRM) mode. BisGMA and UDMA quantification was performed in single ion monitoring (SIM) mode. The acquisition parameters are described in Table 3 and Table 4. All measurements were performed in duplicate.

The calibration curve of dBPA consisted of 15 concentrations from 0.003 to 50 ng/mL, while the calibration curve of UDMA and BisGMA included 16 concentrations from 0.003 to 100 ng/mL. Acetonitrile/water (40:60 (*v*/*v*)) was used as a dilution solvent for BPA analysis and methanol/water (50:50 (*v*/*v*)) was used for UDMA/BisGMA analysis. The limit of detection (LOD) and the limit of quantification (LOQ) were calculated based on the standard deviation of the blank as described by Wenzl et al. [42]. Calibration was validated by the distribution of data points on the residual plot and the coefficient of determination r^2^. A uniform residual plot with a coefficient of determination ≥0.95 was taken as evidence for the linearity of the calibration. The calibration for all analytes was linear within the calibration range and the coefficient of determination (r^2^) exceeded the value of 0.99 in all the calibrations. The LOD and LOQ are shown in Table 5.

### 2.4. Statistics

Statistical analyses including graphical processing were performed using Microsoft Excel (Microsoft, Redmond, WA, USA) and R version 3.6.1 (R Development Core Team, R Foundation for Statistical Computing, Wien, Austria). Statistical tests were used to determine significant differences between the materials. Following a Shapiro-Wilk test to ensure normal distribution and a Levene’s test to check for variance homogeneity, a one-way ANOVA followed by a Tukey post hoc test was performed. For non-normal distribution or variance heterogeneity, a Kruskal-Wallis test followed by a Dunn-Bonferroni post hoc test was applied. The significance level was set to 0.05.

## 3. Results

In the present investigation, the average concentrations of monomer elution appeared to be material-dependent and a high degree of variability across materials was observed (see Table 6).

**Figure 2:**
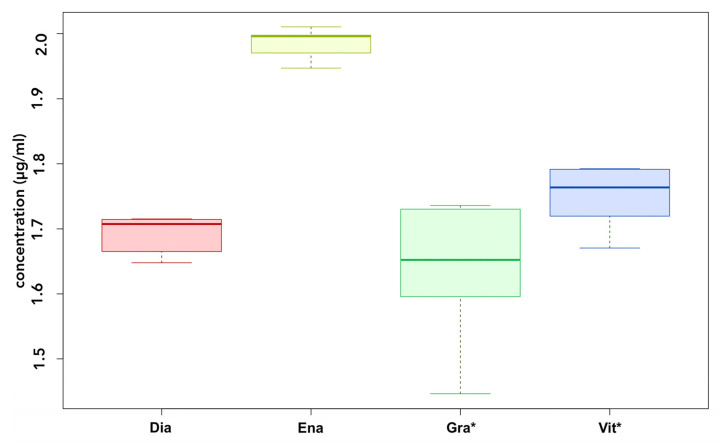
UDMA release after 24 h of incubation. Dia: Dialog Vario; Ena: Enamel Plus; Gra: GC Gradia; Vit: VITA VM LC; * UDMA-based.

**Figure 3:**
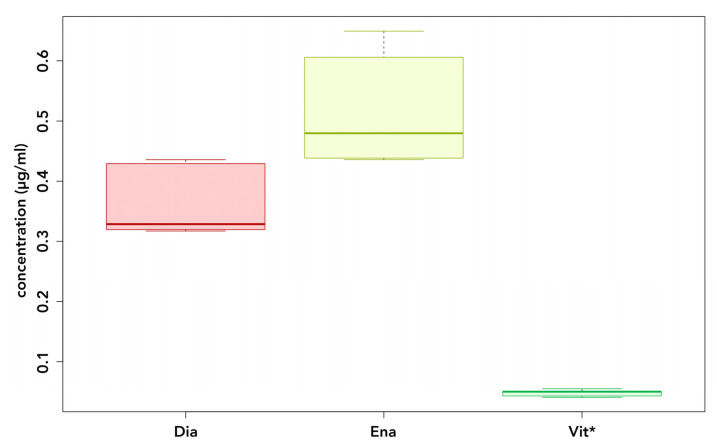
BisGMA release after 24 h of incubation. Dia: Dialog Vario; Ena: Enamel Plus; Vit: VITA VM LC; * UDMA-based.

**Figure 4:**
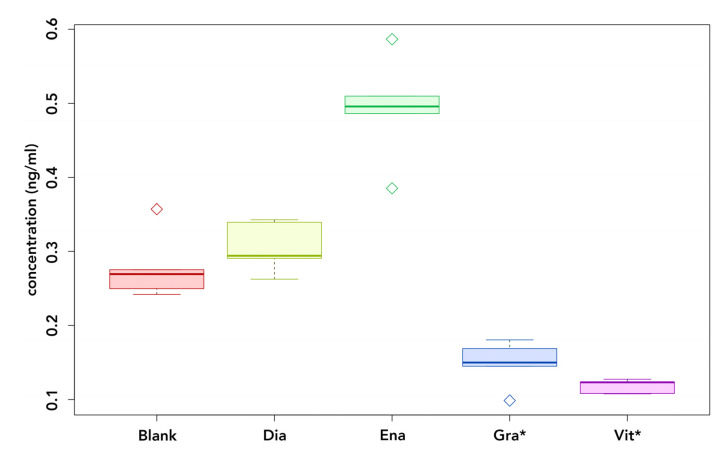
BPA release after 24 h of incubation. Blank: Blank HPLC-grade water blanks; Dia: Dialog Vario; Ena: Enamel Plus; Gra: GC Gradia; Vit: VITA VM LC; * UDMA-based.

Whereas UDMA and/or BisGMA were detectable in the eluates of all materials after 24 h of incubation (see Figure 2 and Figure 3), BPA levels compared to the blanks were only elevated in the eluates of conventional materials (see Figure 4).

Separately for each indirect veneering material, the results were as follows:

### 3.1. Conventional Indirect Veneering Composites

Both UDMA and BisGMA were detectable and quantifiable in the eluates of Enamel Plus HFO and Dialog Vario. After subtracting the BPA concentrations of HPLC-grade water blanks, BPA levels were elevated in the eluates of Enamel Plus HFO and Dialog Vario. On average, BPA levels were elevated by 0.214 ng/mL in Enamel Plus eluates and by 0.027 ng/mL in Dialog Vario eluates. Regarding all the substances investigated, the greatest elution was shown from Enamel Plus HFO.

### 3.2. UDMA-Based Indirect Veneering Composites

UDMA was quantifiable in the eluates of GC Gradia and Vita VM LC, whereas the latter released higher monomer amounts. Low concentrations of BisGMA were only measurable in Vita VM LC eluates. BPA levels of both materials were below the concentrations of the HPLC-grade water blanks and therefore clinically not relevant.

### 3.3. Statistics

For the statistical analysis of UDMA and BisGMA elution blank samples were excluded because both substances were not detectable in the water blanks. Since BisGMA was not measurable in the eluates of GC Gradia, it was assumed that it was not released, and consequently, in these cases, we assigned a value of 0 for subsequent statistical evaluation.

Normal distributions were found in all groups regarding the release of UDMA and BPA. Regarding elution of BisGMA normal distributions were refuted across all groups. Variance homogeneity for the release of all measured substances was confirmed by a Levene test. Analysis of variance revealed highly significant differences between the indirect veneering materials for the release of UDMA (*p* < 0.001) as well as BPA (*p* < 0.001) and a Kruskal-Wallis test showed highly significant differences concerning BisGMA elution (*p* < 0.001). A pairwise comparison was performed using Tukey’s post-hoc or Dunn-Bonferroni post hoc test, respectively (see Table 6).

Compared to the blank samples, the elevation of BPA concentrations in the eluates of Enamel Plus HFO was significant (*p* < 0.001). Concentrations were elevated by an average of 0.214 ng/mL. In contrast, BPA levels of Dialog Vario eluates were not significantly elevated in comparison to the blanks (*p* = 0.861). Enamel Plus HFO released significantly more BPA than Dialog Vario (*p* < 0.001).

UDMA was found in all the samples, whereas the release from Enamel Plus HFO was significantly higher than from any other material (Dialog Vario *p* < 0.001; VITA VM LC *p* < 0.001; GC Gradia *p* < 0.001). The differences between VITA VM LC and GC Gradia (*p* = 0.07), GC Gradia and Dialog Vario (*p* = 0.55) as well as Dialog Vario and VITA VM LC (*p* = 0.55) were not significant.

Both Dialog Vario (*p* = 0.042) and Enamel Plus HFO (*p* < 0.001) released significantly more BisGMA than GC Gradia, although the difference between them was not significant (*p* = 1.0). Enamel Plus HFO samples released significantly more BisGMA than VITA VM LC samples (*p* = 0.042). No statistically significant findings were made when comparing VITA VM LC to GC Gradia (*p* = 1.0) or Dialog Vario (*p* = 1.0).

## 4. Discussion

In the present study, the elution of UDMA, BisGMA, and BPA from conventional and UDMA-based indirect veneering composites was investigated after 24 h of incubation. In contrast to previously studied direct materials, indirect veneering composites are used in larger quantities but are processed outside the oral cavity, which might lead to reduced monomer elution. This pilot study was intended to assess potential monomer release and to evaluate the impact of different veneering composite compositions (conventional/UDMA-based). An incubation period of 24 h was chosen because current literature showed a logistic monomer release from composites, with the maximum being reached after this period [43,44,45,46]. Furthermore, it is considered the reference period for meta-analysis [47]. Unfortunately, a comparative analysis of the present results with literature data is impeded due to the different material compositions and inconsistent extraction ratios used in the literature [47]. It is recommended to ensure reliability and comparability of the extraction ratio specified in ISO 10993-12, which is widely recognized by international regulatory agencies [47].

In the present study, we were able to assess the clinical significance of monomer elution by using this extraction ratio and relating the surface size of our samples to that of common restorations (see Table 7).

Study results show that UDMA was released from all four veneering composites. Significant differences were found, but contrary to a previous study, most UDMA was released from Enamel Plus HFO, a conventional composite, and not from an UDMA-based material [29]. Comparing the measured UDMA concentrations with values from the literature, despite poor comparability, they appear relatively low [48,49,50]. This is likely due to the use of a laboratory light-curing unit instead of a handheld device, as the light source strongly influences monomer elution [51]. To evaluate the potentially harmful impact of these UDMA levels, a comparison to cytotoxic and genotoxic concentrations seems to be necessary. In this context, the cytotoxic effective concentration of dental monomers is expressed as the TC_50_-concentration [52,53], which is usually determined after 24 h or 48 h monomer exposure on different cell lines [54,55]. Reported TC_50_-concentrations of human gingival fibroblasts after 24 h of UDMA exposure are ranging between 49.88 (±2.35) and 94.11 (±47.06) µg/mL [13,56,57]. It appears unlikely, that eluted monomer concentrations even from large restorations reveal cytotoxic effects when comparing literature data with the measurements of the present study (Table 4 and Table 5).

In contrast, mutagenic effects have been demonstrated even below the respective TC_50_-concentration [58,59,60]. Concentrations at which mutagenic effects occur, such as double-strand breaks, deletions of DNA segments, or the induction of micronuclei, are dependent on the exposed cell and monomer [61,62]. Using a comet assay with human parotid gland cells and lymphocytes, initial DNA damage was observed after 60 min of exposure to UDMA and a concentration of 0.047 µg/mL [63,64]. After six hours of exposure, a relevant number of double-strand breaks in human gingival fibroblasts was detected at a concentration of 14.12 µg/mL [65]. These genotoxic effects were confirmed in vivo using the eluates of UDMA-based composites [66]. In contrast, an older study by Schweikl et al. found only a slight increase of micronuclei compared to the control group after UDMA exposure [67]. While it seems unlikely, genotoxic effects cannot be ruled out as the concentrations reported in the literature vary widely and have been reported even after short exposition times. For further clarification, in vitro or in vivo studies with the eluates of the investigated materials are required.

BisGMA was released in low concentrations, but concentrations were elevated in conventional materials. While BisGMA is not part of the composition of the UDMA-based material VITA VM LC, as stated by the manufacturer’s information, we detected small amounts in the eluates. This is not an uncommon finding, since manufacturers are not obliged to disclose the full composition of their materials as this is considered a trade secret [9,68]. Overall, we found only low concentrations, but this is already known in the literature, as BisGMA is either not released or eluted in small quantities in aqueous media like water, artificial saliva, or collected saliva [39,69,70]. Cytotoxic concentrations of BisGMA are reported between 12.41 and 184.53 µg/mL [13,56,57,58,71] and compared to other dental monomers, BisGMA causes the most DNA double-strand breaks with a significant induction at 46.133 µg/mL [12,65]. Depending on the restoration size and the material used, a release of between 0.04 to 0.711 µg/mL BisGMA can be expected after 24 h (see Table 7). While in vivo elution might be higher due to salivary flow and intraoral degradation by abrasion, erosion, and enzymes of the saliva [49], these dynamics are often not taken into account in cytotoxicity tests [72]. Mostly, the constant salivary flow is not considered, because a standing non-renewing cell culture medium is normally used [55,73]. However, in vivo released monomers are cleared early, therefore the measured concentrations can only occur permanently in confined spaces, such as deep cavities [74]. Considering that monomers are cleared early in vivo and that the materials studied are not used in confined spaces, cytotoxic or genotoxic effects due to BisGMA elution also seem unlikely.

BPA concentrations of UDMA-based samples were below concentrations of the pure HPLC-grade water blanks and were therefore considered clinically not relevant. The eluates of both conventional materials (Enamel Plus HFO and Dialog Vario) contained more BPA than the blanks. Many studies on the BPA elution from dental resins did not report concentrations of the blanks [75,76,77] even though the omnipresence of BPA is well known [78,79,80]. Therefore, rules of good practices for BPA analysis recommend considering BPA concentrations of blanks to rule out extraneous BPA [81,82]. In the present study, blank samples of the extraction medium were analyzed together with the eluates from the materials investigated. We found that concentrations were elevated in Enamel Plus HFO eluates by an average of 0.214 ng/mL and in Dialog Vario eluates by 0.027 ng/mL. However, the difference to blank samples was only significant for Enamel Plus eluates, thus the release from Dialog Vario samples may not be clinically relevant. Considering the latest research, the EFSA (European Food Safety Agent) agreed on a Tolerable Daily Intake (TDI) of BPA of 4 µg/kg body weight/day in 2015 [83]. However, in the most recent draft of the re-evaluation of BPA, the EFSA has lower the TDI to 0.04 ng/kg of body weight/day [84]. Due to its omnipresence, BPA can even be detected in human urine and reports from the current literature estimate daily intakes just below 0.1 µg/kg body weight/day [85,86,87]. Therefore, daily BPA exposure is often already above the TDI. Considering the latest recommendation of the EFSA, the BPA exposure from indirect veneering composites and other dental materials should be reduced by using BPA-free materials. Estrogenic activity cannot be ruled out, particularly when placing restorations veneered with Enamel Plus HFO. Further studies with the eluates of Enamel Plus HFO using estrogen-sensitive cell culture systems are required for a definitive evaluation of the local effect of this material. In conclusion, UDMA-based materials might be the safer choice in terms of BPA exposure.

The major difficulty of in vitro studies is the consideration of all influencing factors of the oral environment, which represents a major limitation of the present work [53]. Immediate immersion after preparation of the samples might have influenced monomer elution. Some studies performed immersion after the post-irradiation cure, usually a storage period of 24 h in the dark [33,50,81,88,89]. This procedure leads to a reduced monomer release [90]. For most materials this procedure is not consistent with the clinical workflow and therefore not recommended [88,91]. However, indirect veneering composites are often stored before insertion. Nevertheless, the authors of this study decided against this procedure in order to evaluate the most extreme release after 24 h in this pilot study. Considering this limitation, in vivo concentrations might be even lower than the reported values of the present study.

Some studies report data from multiple incubation periods of up to one year [44,82,92,93,94]. However, many factors need to be considered when studying long incubation periods. Passive hydrolysis reactions lead to the degradation of monomers in water [95]. Passive and/or enzyme-catalyzed hydrolysis, such as in collected saliva, breaks the ester bonds of the methacrylate groups of BisGMA or UDMA [96,97,98,99]. The hydrolysis of dental monomers often takes place incompletely so that molecules with a different number of cleaved methacrylate groups may be present simultaneously [98,100]. Each of these hydrolysis products has different chemical properties and molar masses, so detection requires adjustment of the analytical method [100,101]. A weekly solvent change, as is often carried out in long-term studies, is not sufficient, as the first degradation processes can already be seen on the first day [47]. Therefore, the analysis of long incubation periods is prone to error and requires a more complex analytical method. We aimed to develop a reliable analytical method to obtain an initial impression of the monomer release from indirect veneering composites and to evaluate the need for follow-up studies. As earlier studies found that maximum elution is reached after 24 h, we chose this incubation time for this study [43,44,45,46]. Nevertheless, the investigation of only one interval and no longer periods is a limitation of this study and future research should examine long-term release, as described above.

Within the limitations of this study, it might be concluded that elution from the veneering composites appears to be material-dependent, but a release of cytotoxic concentrations of UDMA or BisGMA is unlikely. Most biocompatibility concerns regarding composites are related to the release of BPA and its derivatives, such as BisGMA. BPA was found in the eluates of conventional materials (Enamel Plus HFO and Dialog Vario). However, compared to blank samples, BPA levels were only significantly elevated in the Enamel Plus eluates. Since the newly recommended TDI for BPA is even lower than the estimated daily intake, these results of the present study are of clinical relevance. BisGMA concentrations were below cytotoxic concentrations. In UDMA-based composites, BPA-derivatives are usually replaced by UDMA to prevent the release of BPA. In this context, GC the manufacturer of GC Gradia advertises its composites explicitly as BPA-free. These adjustments to the polymer matrix did not lead to increased UDMA levels but lower BisGMA and BPA concentrations. These findings are in contrast to a previous study on temporary crown and bridge materials, which found a significantly increased UDMA release in BPA-free composites, while all BisGMA levels were below the LOD [29]. This might have been caused by the use of a less sensitive analytical method compared to the present study. Due to higher reactivity and lower viscosity, UDMA-based composites show higher degrees of conversion than BisGMA-based composites [102]. Therefore, UDMA-based composites might have particularly benefited from using a laboratory light-curing unit, which could have caused the low UDMA found in this study. Further research on the factors influencing the conversion of conventional and UDMA-based/BPA-free materials is necessary.

Considering the results of this study, the replacement of BPA-derivatives with UDMA in the materials studied may have been beneficial in terms of biocompatibility. In addition to monomer release, other properties must also be considered to evaluate different composite compositions. In this context, the measurement of double bond conversion is a good indicator for the prospective physical properties of a composite system [103]. UDMA molecules are more chemically reactive than BisGMA molecules due to higher molecular flexibility and chain transfer reactions through the -NH group [104]. These properties lead to a greater degree of crosslinking [103,105], allowing a lower co-monomer content and still achieving high conversion with lower polymerization stress compared to BisGMA-based composites [106]. However, UDMA-based dental composites exhibit higher volumetric shrinkage and shrinkage stress than Bis-GMA-based composites [106,107], thus leading to more brittle mechanical characteristics [108]. Therefore, the specific monomer composition of dental resins is tailored to the area of application and may even include both base monomers [109,110].

## 5. Conclusions

Indirect veneering composites elute BPA, UDMA, and BisGMA in aqueous media. Significantly elevated BPA levels compared to blank samples were found in the eluates of one conventional material. Measured BPA concentrations were below the TDI, but considering the estimated daily intake, the TDI could be exceeded. Therefore, an estrogen-like effect of the conventional materials studied in the present investigation cannot be ruled out. Follow-up studies using estrogen-sensitive cell culture systems are necessary. Furthermore, UDMA-based veneering composites elute less BisGMA than conventional composites. Regarding the UDMA elution, no connection to the composition of the polymer matrix (conventional/UDMA-based material) was found. Even after relating the present data to common restoration sizes, the elution of cytotoxic monomer concentrations is unlikely. Since lower BisGMA levels and no BPA were found in UDMA-based materials, the replacement of BPA-derivatives with UDMA may have been beneficial in terms of biocompatibility.

## Figures and Tables

**Table 1:** Monomer composition of the indirect veneering composites tested.

Material	Shade	Main Monomers *	Manufacturer
VITAVM LC	A2	UDMA (CAS: 72869-86-4), TEGDMA (CAS: 109-16-0)	VITA Zahnfabrik, Bad Säckingen, Germany
Dialog Vario	A2	UDMA, 1,4-Butandioldimethacrylat, BisGMA	Schütz Dental, Rosbach, Germany
GC Gradia	A2	UDMA (CAS: 72869-86-4), 2,2-Dimethyl-1,3-propanediyl bismethacrylat (CAS: 1985-51-9), 1,3,5-Triazine-2,4,6-triamine, polymer with formaldehyde (CAS: 9003-08-1)	GC Corporation, Tokyo, Japan
Enamel Plus HFO	A2	1,4-Butandioldimethacrylat, UDMA, BisGMA	Micerium, Avegno, Italy

* According to manufacturers’ information, CAS numbers are given when specified.

**Table 2:** Reference substances.

Name	Abbreviation	Manufacturer	Molecular Mass [g/mol]	CAS-Nr.	Purity
Urethane dimethacrylate	UDMA	Sigma Aldrich, Munich, Germany	470.56	72869-86-4	>97%
Triethylene glycol dimethacrylate	TEGDMA	Sigma Aldrich, Munich, Germany	286.32	109-16-0	99%
Bisphenol A	BPA	Sigma Aldrich, Munich, Germany	228.29	80-05-7	≥99%
Bisphenol A-glycidyl methacrylate	BisGMA	Sigma Aldrich, Munich, Germany	512.59	1565-94-2	not specified
Pyridine-3-sulfonyl chloride	PSC	Sigma Aldrich, Munich, Germany	177.61	16133-25-8	≥98.0%
Bisphenol A-d16	d16BPA	Sigma Aldrich, Munich, Germany	244.38	96210-87-6	98 atom % D

**Table 3:** Acquisition Parameters for BPA and its derivatives using multiple reactions monitoring (MRM) mode.

Compound	Polarity	Parent Ion	Product Ions	Collision Energy [V]	Fragmentor [V]	Cell Accelerator Voltage [V]
BPA	Negative	227	212	28	110	4
			133	28		
d16BPA	Negative	241	223	15	115	
			141	30		
BPA-diPS	Positive	511	354	35	163	
			290	35		
			276	30		
d16BPA-diPS	Positive	525	365	35	170	
			301	40		
			286	30		

diPS: derivatized with pyridine-3-sulfonyl chloride.

**Table 4:** Acquisition Parameters for BisGMA and UDMA using single ion monitoring (SIM) mode.

Compound	Polarity	Parent Ion	Fragmentor [V]	Cell Accelerator Voltage [V]
BisGMA	Positive	535.5	166	4
UDMA	Positive	493.3	170	

**Table 5:** Limits of detection and quantification.

Substance	Limit of Detection	Limit of Quantification
UDMA	0.26 ng/mL	0.84 ng/mL
BisGMA	0.14 ng/mL	0.47 ng/mL
BPA-diPS	0.03 ng/mL	0.09 ng/mL

diPS: derivatized with pyridine-3-sulfonyl chloride.

**Table 6:** Average released monomer concentrations and standard deviations after 24 h of incubation.

Material	BisGMA [µg/mL]	BPA [ng/mL]	UDMA [µg/mL]
VITAVM LC	0.048 ± 0.006 ^ab^	0.118 ± 0.009 ^a^	1.748 ± 0.052 ^a^
Dialog Vario	0.366 ± 0.061 ^ac^	0.306 ± 0.034 ^b^	1.690 ± 0.031 ^a^
GC Gradia	<LOQ ^b^	0.148 ± 0.031 ^a^	1.632 ± 0.119 ^a^
Enamel Plus HFO	0.354 ± 0.067 ^c^	0.493 ± 0.072 ^c^	1.984 ± 0.025 ^b^
Blanks	<LOD	0.279 ± 0.046 ^b^	<LOD

LOD UDMA: 0.26 ng/mL; LOQ UDMA: 0.84 ng/mL; LOD BisGMA: 0.14 ng/mL; LOQ BisGMA: 0.47 ng/mL; LOD BPA: 0.03 ng/mL; LOQ BPA: 0.09 ng/mL; the different superscript letters indicate statistically significant differences within the vertical line (*p* < 0.05).

**Table 7:** Average released monomer concentrations in relation to the most common restorations (after 24 h of incubation). Surface areas (Crown: 3.15 cm^2^; Bridge: 7.32 cm^2^) were calculated using the average surface area of the corresponding crowns of natural teeth [38].

Material	Restoration	BisGMA [µg/mL]	UDMA [µg/mL]	BPA [ng/mL]
VITAVM LC	Crown (first molar)	0.040 ± 0.005	1.461 ± 0.043	-
	Bridge (second premolar to second molar)	0.092 ± 0.120	3.394 ± 0.101	-
Dialog Vario	Crown (first molar)	0.306 ± 0.051	1.413 ± 0.026	-
	Bridge (second premolar to second molar)	0.711 ± 0.118	3.282 ± 0.060	-
GC Gradia	Crown (first molar)	<LOQ	1.364 ± 0.99	-
	Bridge (second premolar to second molar)	<LOQ	3.169 ± 0.231	-
Enamel Plus HFO	Crown (first molar)	0.296 ± 0.056	1.659 ± 0.021	0.179
	Bridge (second premolar to second molar)	0.687 ± 0.130	3.853 ± 4.86	0.416

LOD UDMA: 0.26 ng/mL; LOQ UDMA: 0.84 ng/mL; LOD BisGMA: 0.14 ng/mL; LOQ BisGMA: 0.47 ng/mL; LOD BPA: 0.03 ng/mL; LOQ BPA: 0.09 ng/mL.

## Data Availability

Not applicable.
